# Humidity Fixed Points of Binary Saturated Aqueous Solutions

**DOI:** 10.6028/jres.081A.011

**Published:** 1977-02-01

**Authors:** Lewis Greenspan

**Affiliations:** Institute for Basic Standards, National Bureau of Standards, Washington, D. C. 20234

**Keywords:** aqueous solution, equilibrium, humidity, relative humidity, salt, saturated salt solution, vapor pressure, water vapor

## Abstract

An evaluated compilation of equilibrium relative humidities in air versus temperature from pure phase to approximately 10^5^ pascal (1 atm) in pressure is presented for 28 binary saturated aqueous solutions. The relative humidities of the solutions range from about 3 to 98 percent. Using a data base from 21 separate investigations comprising 1106 individual measurements, fits were made by the method of least squares to regular polynomial equations with two through four coefficients. Equations and tables are presented along with the estimated uncertainties in the correlated results.

## 1. Introduction

Research, hygrometer calibration, testing and material conditioning often require the accurate control of humidity in a working space. The common methods of controlling the humidity accurately use either a humidity generator [1A]^1^ or the equilibration of a closed space with a chemical system [1B] which produces the desired equilibrium vapor pressure.

Humidity generators tend to be expensive and complex whereas equilibration with chemical systems that provide fixed points is a relatively inexpensive and simple method of humidity control. Among the chemical systems used for this purpose are aqueous sulphuric acid solutions, glycerine and water solutions and single and binary salt solutions. Each such solution offers a degree of humidity adjustment that can be achieved by changing its concentration. On the other hand, special problems are associated with the use of solutions because their concentrations must be measured and controlled. Not only must the concentration of the solution be determined initially but the presence of any humidity sources or sinks in the controlled space and even the initial equilibration process of the space can alter the solution concentration.

An especially useful method of humidity control by chemical system involves the use of binary saturated aqueous solutions (primarily of single salts) in which the solute is highly non-volatile.

At any temperature, the concentration of a saturated solution is fixed and does not have to be determined. By providing excess solute, the solution will remain saturated even in the presence of modest sources or sinks. Where the solute is a solid in the pure phase, it is easy to determine that there is indeed saturation. Due to the ease of its use, this is a popular method of humidity control.

Since a given saturated salt solution provides only one relative humidity (RH) at any desired temperature, a different relative humidity must be achieved by selecting another appropriate salt. Though much data on saturated salt solutions have been produced and many compilations of the equilibrium relative humidities of selected saturated salt solutions exist, there are no compilations for which the data have been critically analyzed and estimates of the uncertainties involved given, a step which is abolutely essential to the implimentation of the concept of fixed points.

We have moved to fill this gap by compiling, from the literature, data on a sufficient variety of saturated salt solutions to cover the entire range of relative humidity at reasonably close intervals. We have adjusted these data [1–21] to be consistent with temperatures on IPTS-68 and the most recent equations for the vapor pressure of water [22]. We have also analyzed the experimental techniques used in obtaining the original data and have made estimates of the uncertainties in the original data. We have then used these data to calculate “best” values of relative humidity in air as a function of temperature from pure phase to approximately 10^5^ pascal (1 atm) in pressure for these saturated solutions.

## 2. Background

The methods used by investigators to determine the water vapor in equilibrium with saturated salt solutions are diverse. A short description of the various methods used in the referenced papers is of interest.

1. *The direct measurement of the vapor pressure*. A chamber containing a saturated salt solution at a controlled temperature is first evacuated to remove all gases. Evaporation from the solution is then allowed to proceed until the ambient vapor, essentially all water, has come to equilibrium with the solution and a direct determination of the total pressure within the chamber is made by conventional pressure measurement techniques.
2. *Dew point measurement*. The dew point of the gas within a chamber containing a saturated salt solution at controlled temperature is measured by means of a cooled mirror within the chamber. Using vapor pressure tables or equations, this dew point is converted to the vapor pressure of water.
3. *Isopiestic vapor pressure measurement*. The vapor pressure of a saturated salt solution in one cell or chamber is allowed to come to equilibrium with a cell or chamber containing a reference solution at a fixed temperature. The reference solution must be well characterized as to vapor pressure as a function of concentration at the reference temperature. Under the equilibrium condition, the equilibrium vapor pressure of the saturated salt solution is identical to the equilibrium vapor pressure of the reference solution. After the two cells have reached equilibrium, the concentration of the reference solution is determined (usually by weighing) and the vapor pressure is calculated.
4. *Relative vapor pressure measurement*. A chamber containing a saturated salt solution and a chamber containing pure water or other well characterized solution are each evacuated to remove all non-water vapor gases. The two chambers are maintained at the same temperature and the absolute pressure of the saturated salt solution is measured as in the first method. In addition the pressure difference between the two chambers and/or the pressure of the reference solution is determined. The ratio of the vapor pressure of the saturated salt solution to the vapor pressure of the water is the activity (or relative humidity) of the saturated salt solution.
5. *Measurement with a calibrated humidity sensor*. A chamber containing a saturated salt solution and a humidity sensor are brought to equilibrium at a controlled temperature. Calibration of the sensor before or/and after the measurement provides the means of determining the equilibrium vapor pressure.
6. *Gravimetric determination*. Dry gas is passed through the binary saturated solution at a fixed temperature. The water vapor in the effluent gas is absorbed by a desiccant and measured by weighing. The volume of the gas is also determined. From these the vapor pressure or the mixing ratio can be determined.

As one would imagine, the errors associated with these methods differ as to source and magnitude. The errors in any of the methods are also functions of the level of vapor pressure being measured as well as the temperature of the saturated salt solution. There is, therefore, probably no one method that gives a best measurement under all conditions.

## 3. Method

We have accumulated experimental data from various researchers [1–21] and calculated “best” values of relative humidity and the associated uncertainties of those values. Typical methods of calculating or recalculating the relative humidity and associated uncertainties for the various investigations are given in the Appendix. Our data base consists of 21 investigations and includes some of the most cited work in the field. In total, 1106 individual calculations of relative humidities and associated uncertainties were made which involved 89 saturated solutions. Not all data nor all saturated solutions in this study were found satisfactory for use.

The original data were corrected to be consistent with temperature on IPTS-68, with the most recent formulation for the vapor pressure of water [22] and with the most recent equations for the enhancement of water vapor in air [23]. The computed relative humidity data were then collated and fitted by the method of least squares to regular polynomials as a function of temperature in degrees Celsius (IPTS-68). In the fitting process, each datum was weighted inversely proportional to the estimated uncertainty of the datum. The order of the polynomial used in the fit was determined by an F-test or by analysis of the result of fits to various orders. An arbitrary decision was made not to use any order higher than 3. Also, no data at temperatures below 0 °C or above 104 °C were used in the fits.

In the fitting process, the standard deviation of the predicted value was computed for each datum. These standard deviations were themselves fitted to a quadratic equation, as a function of temperature, by the method of least squares. At any desired temperature for a given saturated salt solution, the standard deviation of the predicted value was calculated using the appropriate quadratic equation. Three times this value was then assigned as the estimated uncertainty for the corresponding value of relative humidity, with certain exceptions discussed below. This is the value which appears in table 2.

Where a number of investigations of the same solution existed and the relative humidity vs temperature results of one investigation were completely inconsistent with the results of the other investigations, the data of the deviant investigation were eliminated and a new fit made.

The data used in this paper met one of the following criteria: (1) a large number of investigations were included and exhibited a small residual standard deviation of the relative humidity vs temperature fits; (2) although few investigations were included, the method of measurement was judged to be superior and estimates of the uncertainties of the original measurements themselves were small; and (3) the data were in a relative humidity range which was not approximated by any of the other binary saturated solutions.

## 4. Results

Table 1 contains coefficients for the data of the selected salts fitted to an equation of the form:

$$RH=\sum_{i=0}^{3}A_{i}t^{i}$$

where *RH* is in percent and *t* is in °C (IPTS-68). The salts are listed in ascending order of *RH* at 25 °C. Also included in table 1 is the residual standard deviation of the fit, the range of temperature over which the fit was performed and references for the fundamental data that were involved in that particular fit.

Table 2 gives the calculated relative humidities for each of the binary saturated solutions at 5-degree intervals along with the estimated uncertainties in relative humidity at each of the temperatures. The saturated salt solutions are presented in the same order as in table 1.

## 5. Discussion

Although the method used for fitting the data gave no problems, the assignment of weights to each datum required some judgment. Three methods of weighting were considered: (1) weights were assigned inversely proportional to the variance of the individual datum where the variance was taken as the square of the total uncertainty; (2) weights were assigned inversely proportional to the estimated total uncertainty of the individual datum; and (3) weights of unity were assigned to all data.

All of the data were fitted three times, once for each type of weighting. The results were assembled into three tables of relative humidity at 5-degree intervals. Each calculated value of relative humidity was assigned an uncertainty equal to three times the standard deviation of the predicted value. As might be expected, the calculated relative humidities and the corresponding uncertainties differed for each of the three weightings. For the saturated solutions chosen for presentation in this paper, it was noted with some satisfaction that all relative humidities calculated from the three differently weighted fits agreed with each other to within the assigned uncertainty for each.

A weighting inversely proportional to the square of the estimated total uncertainty for each datum was judged to be inappropriate. Although it is common to assign weights proportional to the inverse of sigma squared such an approach is usually based on a sigma which is statistically determined. This is not the case here. The method used to obtain the estimated total uncertainty is given in the Appendix. It was felt that the use of the square of the estimated uncertainty would have placed an unacceptably high value on the author’s estimate of the errors contributing to the total uncertainty. Some investigators did not provide sufficient information in their publications to make possible completely objective estimates of their errors. In those cases, the estimated total uncertainty included components based on the author’s subjective judgments.

A weighting of unity was likewise unsatisfactory since it would in no way take into account the innate difference in uncertainty due to method, temperature and relative humidity range, nor would it place any reliance on the author’s judgment of the quality of the research. A weighting proportional to the inverse of the estimated uncertainty appeared to be a reasonable compromise between the other extremes and all data presented in this publication were processed using that weighting method.

Where the data for a particular saturated salt solution included a number of investigations, three times the standard deviations of the computed values were accepted as the estimated uncertainty. Where the data were based only on one or two investigations it is evident that self consistent data, though quite inaccurate, could give small estimated standard deviations of the computed values. It is also evident that such standard deviations are not a valid estimate of uncertainty. Under those circumstances where the results from fitting the polynomial equation to the original data for any saturated salt solution gave values for three times the standard deviation of the predicted value that were less than the *estimated* total uncertainty of the original data, it was the estimated total uncertainty of the original data which was used as the final estimate of uncertainty for the calculated “best” value of relative humidity.

The data presented in table 2 are given at 5 °C intervals over the temperature range of the original data with extrapolations beyond these ranges never exceeding 2.5 °C. All calculated values of relative humidity are given to 0.01 percent relative humidity. This does not in any way imply an accuracy of 0.01 percent. The designated estimated uncertainties still give the best prediction of accuracy. It was felt that to fail to give the relative humidities to .01 percent would be discarding information, imprecise as it might be. Since the estimated uncertainties are given, we see no problem with presenting the values of relative humidity with figures far beyond their estimated uncertainties.

The uncertainties presented do not include uncertainties in the vapor pressure equation [22] or enhancement equations [23] used. The results presented are therefore for the exact values of references [22] and [23]. The enhancement factor for a saturated salt solution in air is not known precisely. Analysis of the factors involved indicate that at one atmosphere pressure or less, the difference between the enhancement factor over a saturated salt solution and over pure water is negligible. That is not the case at high pressures. The data presented are therefore considered valid near or below one atmosphere total pressure. If saturation vapor pressure values other than those given by Wexler [22] are used, the relative humidities should be multiplied by the ratio of these saturation vapor pressures to those of Wexler.

Many compilations of non-critically evaluated data on the equilibrium humidity of saturated salt solutions exist [24–38]. Table 3 is a comparison of values from this work and corresponding values taken from five of these other compilations at four temperatures. Of the listed compilations, only this work (column a) and Hickman’s work (column d) give the sources of the data. Hickman’s values (in column d) were directly copied from his cited references without modification. None of the compilations other than ours (column a) gives estimates of uncertainty. Therefore, one would logically conclude that the authors of those compilations consider their values to be uncertain only in the last figure presented. It is also likely that some of the values in one compilation came from the same sources as the values in other compilations — such a relationship appears to exist between column b and column d.

If we assume an uncertainty of 1/2 of the last digit in the values given in these other compilations, and if we add that uncertainty to the estimated uncertainty for the corresponding values in column a, we find that the values in column a (the results of this work) agree with the values in at least one of the other compilations to within this composite uncertainty at all points, except for:

Potassium carbonate at 10 °C
Sodium bromide at 20 °C
Ammonium chloride at 30 °C
Potassium bromide at 10 °C, 20 °C, and 30 °C
Potassium chloride at 10 °C, and 20 °C

It should be noted that this comparison of compilations is over a limited temperature range and for only 17 of the 28 salt solutions evaluated and collated in this paper.

## Figures and Tables

**Table 1:** Summary of Least Squares Fits to $RH=\sum_{i=0}^{3}A_{i}t^{i}$ for Selected Saturated Salt Solutions

Salt	*A*_0_	*A*_1_	*A*_2_	*A*_3_	*σ*	No. of Points	*t*_min_	*t*_max_	Data Source (a)

Cesium Fluoride	6.20938	−0.143381	0.123037 × 10^−2^		0.54	21	5.0	80.0	2, 17
Lithium Bromide	7.75437	−0.0654994	0.420737 × 10^−3^		.22	21	0.0	100.0	2, 14
Zinc Bromide	9.28455	−0.0906508	0.118143 × 10^−2^		.20	16	5.0	70.0	2, 14
Potassium Hydroxide	16.7049	−0.511352	0.796712 × 10^−2^	−0.426364 × 10^4^	.28	14	5.0	70.0	2
Sodium Hydroxide	11.5581	−0.132339			.90	24	15.0	75.0	1, 16
Lithium Chloride	11.2323	−0.00824245	−0.214890 × 10^−3^		.67	100	0.0	100.3	1, 3, 10, 11, 17, 18, 20
Calcium Bromide	23.5670	−0.136117	−0.585836 × 10^−2^		.06	7	11.2	25.0	11, 14
Lithium Iodide	22.8216	−0:232642	0.132306 × 10^−2^	−0.168738 × 10^−4^	.05	15	5.0	70.0	2, 14
Potassium Acetate	22.4388	0.156288	0.612868 × 10^−2^		.22	10	11.2	31.0	11, 14, 17
Potassium Fluoride	65.7907	−2.07303	0.305676 × 10^−1^		.36	8	25.0	90.0	6, 14
Magnesium Chloride	33.6686	−0.00797397	−0.108988 × 10^−2^		.28	48	0.00	99.4	1, 7, 11, 14, 17, 19, 21
Sodium Iodide	42.6040	0.00854045	−0.933320 × 10^−2^	0.761055 × 10^−4^	.50	25	5.0	90.0	1, 6, 11, 14
Potassium Carbonate	43.1315	0.00147523			.31	9	0.0	30.0	8, 14, 17
Magnesium Nitrate	60.3514	−0.298153			.34	24	0.0	48.1	1, 8, 17, 19, 21
Sodium Bromide	64.7190	−0.221990	−0.402414 × 10^−2^	0.0590331 × 10^−4^	.44	22	5.0	80.0	2, 6, 11, 17
Cobalt Chloride	73.0330	0.0852795	−0.218455 × 10^−1^	0.218691 × 10^−^	1.44	17	23.4	78.9	6
Potassium Iodide	74.5466	−0.253167	0.104383 × 10^−2^		0.20	12	5.0	90.0	1, 6
Strontium Chloride	78.5322	−0.273114	−0.135136 × 10^−2^		.02	7	5.0	30.0	1, 17
Sodium Nitrate	79.5738	−0.193192	−0.122102 × 10^−2^	0.174308 × 10^−4^	.37	25	5.0	90.0	1, 6, 9, 17
Sodium Chloride	75.5164	0.0398321	−0.265459 × 10^−2^	0.284800 × 10^−4^	.21	44	0.0	80.0	1, 6, 8, 11, 12, 13, 15, 17, 19, 21
Ammonium Chloride	81.8777	−0.132271			.60	20	11.2	31.0	9, 11
Potassium Bromide	86.6424	−0.332271	0.459734 × 10^−2^	−0.199429 × 10^−4^	.14	11	5.0	80.0	1, 6, 17
Ammonium Sulfate	81.7794	−0.0715320			.40	21	0.4	48.0	9, 18, 20
Potassium Chloride	88.6190	−0.193340	0.899706 × 10^−3^		.45	39	0.0	90.0	1, 6. 8, 9, 12, 17
Strontium Nitrate	94.2127	−0.366025			.19	5	5.0	25.0	1
Potassium Nitrate	96.3361	0.0112371	−0.484514 × 10^−2^		.80	22	0.6	48.1	9, 17, 19, 21
Potassium Sulfate	98.7792	−0.0590502			.47	18	0.5	52.3	12, 19, 21
Potassium Chromate	103.934	−0.310163	0.273023 × 10^−2^		.22	7	23.7	50.8	12, 17

(a) Numbers correspond to references.

**Table 2:** Equilibrium Relative Humidity of Selected Saturated Salt Solutions from 0 to 100 °C

T °C	Relative Humidity, %									
	Cesium Fluoride	Lithium Bromide	Zinc Bromide	Potassium Hydroxide	Sodium Hydroxide	Lithium Chloride	Calcium Bromide	Lithium Iodide	Potassium Acetate	Potassium Fluoride

0		7.75 ± 0.83				11.23 ± 0.54				
5	5.52 ± 1.9	7.43 ± 0.76	8.86 ± 0.89	14.34 ± 1.7		11.26 ± 0.47		21.68 ± 0.30		
10	4.89 ± 1.6	7.14 ± 0.69	8.49 ± 0.74	12.34 ± 1.4		11.29 ± 0.41	21.62 ± 0.50	20.61 ± 0.25	23.38 ± 0.53	
15	4.33 ± 1.4	6.86 ± 0.63	8.19 ± 0.61	10.68 ±1.1	9.57 ± 2.8	11.30 ± 0.35	20.20 ± 0.50	19.57 ± 0.20	23.40 ± 0.32	
20	3.83 ± 1.1	6.61 ± 0.58	7.94 ± 0.49	9.32 ± 0.90	8.91 ± 2.4	11.31 ± 0.31	18.50 ± 0.50	18.56 ± 0.16	23.11 ± 0.25	
25	3.39 ± 0.94	6.37 ± 0.52	7.75 ± 0.39	8.23 ± 0.72	8.24 ± 2.1	11.30 ± 0.27	16.50 ± 0.20	17.56 ± 0.13	22.51 ± 0.32	30.85 ± 1.3
30	3.01 ±0.77	6.16 ± 0.47	7.62 ± 0.31	7.38 ± 0.56	7.58 ± 1.7	11.28 ± 0.24		16.57 ± 0.10	21.61 ± 0.53	27.27 ± 1.1
35	2.69 ± 0.63	5.97 ± 0.43	7.55 ± 0.25	6.73 ± 0.44	6.92 ± 1.5	11.25 ± 0.22		15.57 ± 0.08		24.59 ± 0.94
40	2.44 ± 0.52	5.80 ± 0.39	7.54 ± 0.20	6.26 ± 0.35	6.26 ± 1.2	11.21 ± 0.21		14.55 ± 0.06		22.68 ± 0.81
45	2.24 ± 0.44	5.65 ± 0.35	7.59 ± 0.17	5.94 ± 0.29	5.60 ± 1.0	11.16 ± 0.21		13.49 ± 0.05		21.46 ± 0.70
50	2.11 ± 0.40	5.53 ± 0.31	7.70 ± 0.16	5.72 ± 0.27	4.94 ± 0.85	11.10 ± 0.22		12.38 ± 0.05		20.80 ± 0.62
55	2.04 ± 0.38	5.42 ± 0.28	7.87 ± 0.17	5.58 ± 0.28	4.27 ± 0.73	11.03 ± 0.23		11.22 ± 0.05		20.60 ± 0.56
60	2.03 ± 0.40	5.33 ± 0.25	8.09 ± 0.19	5.49 ± 0.32	3.61 ± 0.65	10.95 ± 0.26		9.98 ± 0.06		20.77 ± 0.53
65	2.08 ± 0.44	5.27 ± 0.23	8.38 ± 0.24	5.41 ± 0.39	2.95 ± 0.60	10.86 ± 0.29		8.65 ± 0.07		21.18 ± 0.53
70	2.20 ± 0.52	5.23 ± 0.21	8.72 ± 0.30	5.32 ± 0.50	2.29 ± 0.60	10.75 ± 0.33		7.23 ± 0.09		21.74 ± 0.56
75	2.37 ± 0.62	5.20 ± 0.19			1.63 ± 0.64	10.64 ± 0.38				22.33 ± 0.61
80	2.61 ± 0.76	5.20 ± 0.18				10.51 ± 0.44				22.85 ± 0.69
85		5.22 ± 0.17				10.38 ± 0.51				23.20 ± 0.80
90		5.26 ± 0.17				10.23 ± 0.59				23.27 ± 0.93
95		5.32 ± 0.16				10.07 ± 0.67				
100		5.41 ± 0.17				9.90 ± 0.77				

T °C	Relative Humidity, %									
	Magnesium Chloride	Sodium Iodide	Potassium Carbonate	Magnesium Nitrate	Sodium Bromide	Cobalt Chloride	Potassium Iodide	Strontium Chloride	Sodium Nitrate	Sodium Chloride

0	33.66 ± 0.33		43.13 ± 0.66	60.35 ± 0.55						75.51 ± 0.34
5	33.60 ± 0.28	42.42 ± 0.99	43.13 ± 0.50	58.86 ± 0.43	63.51 ± 0.72		73.30 ± 0.34	77.13 ± 0.12	78.57 ± 0.52	75.65 ± 0.27
10	33.47 ± 0.24	41.83 ± 0.83	43.14 ± 0.39	57.36 ± 0.33	62.15 ± 0.60		72.11 ± 0.31	75.66 ± 0.09	77.53 ± 0.45	75.67 ± 0.22
15	33.30 ± 0.21	40.88 ± 0.70	43.15 ± 0.33	55.87 ± 0.27	60.68 ± 0.51		70.98 ± 0.28	74.13 ± 0.06	76.46 ± 0.39	75.61 ± 0.18
20	33.07 ± 0.18	39.65 ± 0.59	43.16 ± 0.33	54.38 ± 0.23	59.14 ± 0.44		69.90 ± 0.26	72.52 ± 0.05	75.36 ± 0.35	75.47 ± 0.14
25	32.78 ± 0.16	38.17 ± 0.50	43.16 ± 0.39	52.89 ± 0.22	57.57 ± 0.40	64.92 ±3.5	68.86 ± 0.24	70.85 ± 0.04	74.25 ± 0.32	75.29 ± 0.12
30	32.44 ± 0.14	36.15 ± 0.43	43.17 ± 0.50	51.40 ± 0.24	56.03 ± 0.38	61.83 ± 2.8	67.89 ± 0.23	69.12 ± 0.03	73.14 ± 0.31	75.09 ±0.11
35	32.05 ± 0.13	34.73 ± 0.39		49.91 ± 0.29	54.55 ± 0.38	58.63 ± 2.2	66.96 ± 0.23		72.06 ± 0.32	74.87 ± 0.12
40	31.60 ± 0.13	32.88 ± 0.37		48.42 ± 0.37	53.17 ± 0.41	55.48 ± 1.8	66.09 ± 0.23		71.00 ± 0.34	74.68 ± 0.13
45	31.10 ± 0.13	31.02 ± 0.37		46.93 ± 0.47	51.95 ± 0.47	52.56 ± 1.5	65.26 ± 0.24		69.99 ± 0.37	74.52 ± 0.16
50	30.54 ± 0.14	29.21 ± 0.40		45.44 ± 0.60	50.93 ± 0.55	50.01 ± 1.4	64.49 ± 0.26		69.04 ± 0.42	74.43 ± 0.19
55	29.93 ± 0.16	27.50 ± 0.45			50.15 ± 0.65	48.02 ± 1.4	63.78 ± 0.28		68.15 ± 0.49	74.41 ± 0.24
60	29.26 ± 0.18	25.95 ± 0.52			49.66 ± 0.78	46.74 ± 1.5	63.11 ± 0.31		67.35 ± 0.57	74.50 ± 0.30
65	28.54 ± 0.21	24.62 ± 0.62			49.49 ± 0.94	46.33 ± 1.9	62.50 ± 0.34		66.64 ± 0.67	74.71 ± 0.37
70	27.77 ± 0.25	23.57 ± 0.74			49.70 ± 1.1	46.97 ± 2.3	61.93 ± 0.38		66.04 ± 0.78	75.06 ± 0.45
75	26.94 ± 0.29	22.85 ± 0.88			50.33 ± 1.3	48.80 ± 2.9	61.43 ± 0.43		65.56 ± 0.91	75.58 ± 0.55
80	26.05 ± 0.34	22.52 ± 1.0			51.43 ± 1.5	52.01 ± 3.7	60.97 ± 0.48		65.22 ±1.1	76.29 ± 0.65
85	25.11 ± 0.39	22.63 ± 1.2					60.56 ± 0.54		65.03 ± 1.2	
90	24.12 ± 0.46	23.25 ± 1.4					60.21 ± 0.61		65.00 ± 1.4	
95	23.07 ± 0.52									
100	21.97 ± 0.60									

T °C	Relative Humidity, %							
	Ammonium Chloride	Potassium Bromide	Ammonium Sulfate	Potassium Chloride	Strontium Nitrate	Potassium Nitrate	Potassium Sulfate	Potassium Chromate

0			82.27 ± 0.90	88.61 ± 0.53		96.33 ±2.9	98.77 ± 1.1	
5		85.09 ± 0.26	82.42 ± 0.68	87.67 ± 0.45	92.38 ± 0.56	96.27 ± 2.1	98.48 ± 0.91	
10	80.55 ± 0.96	83.75 ± 0.24	82.06 ± 0.51	86.77 ± 0.39	90.55 ± 0.38	95.96 ± 1.4	98.18 ± 0.76	
15	79.89 ± 0.59	82.62 ± 0.22	81.70 ± 0.38	85.92 ± 0.33	88.72 ± 0.28	95.41 ± 0.96	97.89 ± 0.63	
20	79.23 ± 0.44	81.67 ± 0.21	81.34 ± 0.31	85.11 ± 0.29	86.89 ± 0.29	94.62 ± 0.66	97.59 ± 0.53	
25	78.57 ± 0.40	80.89 ± 0.21	80.99 ± 0.28	84.34 ± 0.26	85.06 ± 0.38	93.58 ± 0.55	97.30 ± 0.45	97.88 ± 0.49
30	77.90 ± 0.57	80.27 ± 0.21	80.63 ± 0.30	83.62 ± 0.25		92.31 ± 0.60	97.00 ± 0.40	97.08 ± 0.41
35		79.78 ± 0.22	80.27 ± 0.37	82.95 ± 0.25		90.79 ± 0.83	96.71 ± 0.38	96.42 ± 0.37
40		79.43 ± 0.24	79.91 ± 0.49	82.32 ± 0.25		89.03 ± 1.2	96.41 ± 0.38	95.89 ± 0.37
45		79.18 ± 0.26	79.56 ± 0.65	81.74 ± 0.28		87.03 ± 1.8	96.12 ± 0.40	95.50 ± 0.40
50		79.02 ± 0.28	79.20 ± 0.87	81.20 **±** 0.31		84.78 ±2.5	95.82 **±** 0.45	95.25 ± 0.48
55		78.95 **±** 0.32		80.70 **±** 0.35				
60		78.94 **±** 0.35		80.25 **±** 0.41				
65		78.99 **±** 0.40		79.85 **±** 0.48				
70		79.07 **±** 0.45		79.49 ± 0.57				
75		79.16 **±** 0.50		79.17 **±** 0.66				
80		79.27 **±** 0.57		78.90 **±** 0.77				
85				78.68 **±** 0.89				
90				78.50 **±** 1.0				
95								
100								

**Table 3:** Comparison of Relative Humidity Values of Selected Saturated Salt Solutions for Various Compilations

Saturated Salt	Relative Humidity, %																				
	10° C				20° C						30 °C						40 °C				
	a	b	c	d	a	b	c	d	e	f	a	b	c	d	e	f	a	b	c	d	e

Potassium Hydroxide	12.34			13	9.32			9			7.38			7			6.26			6	
Lithium Chloride	11.29	14		13	11.31	12		12		15	11.28	12		12			11.21	11		11	
Potassium Acetate	23.38	21		24	23.11	22		23		20	21.61	22		22							
Magnesium Chloride	33.47	34	34.7	34	33.07	33	33.1	33	33		32.44	33	31.7	33	32		31.60	32	31.3	32	32
Sodium Iodide	41.83			42	39.65			39			36.15			36			32.88			33	
Potassium Carbonate	43.14	47	47.0	47	43.16	44	44.0	44	42		43.17	43	43.0	43							
Magnesium Nitrate	57.36	57		57	54.38	55		55			51.40	52		52			48.42	49		49	
Sodium Bromide					59.14					58											
Cobalt Chloride											61.83		61.9				55.48		56.6		
Potassium Iodide	72.11			72	69.90			70			67.89			68							
Sodium Chloride	75.67	76	76.9	76	75.47	76	75.8	76	76		75.09	75	75.1	75	75		74.68	75	74.4	75	75
Ammonium Chloride					79.23				79.2	79.2	77.90				79.5	79.5					
Potassium Bromide	83.75		86.0		81.67		84.0			84	80.27		82.0				79.43		80.0		
Ammonium Sulfate	82.06	82		82	81.34	81		81	81	81.0	80.63	80		80	81.1	81.1	79.91	79		79	81.1
Potassium Chloride	86.77	88	87.4	88	85.11	86	86.3	86	86		83.62	85	84.5	84	84		82.32	82	82.8	82	83
Potassium Nitrate	95.96	95	95.1	95	94.62	93	94.2	93			92.31	91	92.5	91			89.03	88	89.4	88	
Potassium Sulfate	98.18	98	98.2	98	97.59	97	97.1	97	96.5		97.00	96	96.6	96	96.5		96.41	96	96.1	96	96.5

a Values from this work.

b Values from references 24 and 25 which are identical.

c Values from reference 26.

d Values from reference 27.

e Values from reference 28.

f Values from reference 29.

## Footnotes

1 Figures in brackets indicate the literature references at the end of this paper.

## 6. Appendix

In all cases, the most fundamental measurements presented were used to calculate the actual relative humidity obtained by each investigator for each datum. No attempt was made to evaluate purity of water or solute or its effect in any investigation.

As a first step, all temperatures were converted from the temperature scale in which the data were presented into IPTS-68 temperature equivalents. Where the temperature scales were not given, a judgment was made as to the most likely temperature scale used, based on the date of the research.

Likewise, where vapor pressures based on vapor pressure equations or tables were given, these were converted to new vapor pressures based on the Wexler formulation. In the case of reported relative humidities based on dew-point measurements, the dew-point temperature was reconstructed from a knowledge of the vapor pressure equation used. From the reported control temperature and the reconstructed dew-point temperature a new relative humidity was calculated using the Wexler and Greenspan equations for vapor pressures and enhancements factors, respectively.

Where the isopiestic method was used with sulfuric acid as the isopiestic solution, the values of Shankman [39] for sulfuric acid activity were used to determine the relative humidity of the saturated salt solution. This was done (1) for consistency, because many of the researchers had done likewise; (2) because Shankman described his experimental work in sufficient detail to enable us to judge its quality and to estimate the uncertainty in his work; and (3) his values appeared to be the most accurate available.

In determining estimates of total uncertainty for each datum, the uncertainty was taken as the square root of the sums of individual uncertainties (in terms of relative humidity) squared as described by Ku [40]. Individual uncertainties involved in the individual measurements were obtained from the investigators’ own estimates where these seemed reasonable. Where the investigator did not present a reasonable estimate of uncertainty for a particular parameter, this author made his own estimate of the uncertainty of that parameter based on his judgment of the investigator’s work and his estimate of the state of the art at the time of the investigation. The relative humidity uncertainty associated with each of the parameter uncertainties was obtained by calculating the relative humidity with and without the uncertainty added to the related parameter, the difference being the relative humidity uncertainty for that particular parameter.

In some cases the individual parameter uncertainties are not independent in their effect on the relative humidity uncertainty. A case in point is the relative vapor pressure measurement method. In this technique, the individual temperature and pressure measurement uncertainties are of no great consequence, it is the estimates of the temperature difference and the *pressure difference* in the two pressure measurements that are significant. In addition, an estimate of the degree of equilibrium achieved is of significance. In these types of situations, estimates of the differences were used in lieu of estimates of the individual measurements.

In the case of the relative humidity sensor calibration technique, an estimate of the calibration uncertainty as well as temperature uncertainty were used. In the isopiestic technqiue, the relevant uncertainties are the temperature difference, the concentration determination, the uncertainty in equilibrium and the uncertainty in the reference solution data.

Composite uncertainties for each datum based on the square root of the sum of the individual parameter uncertainties squared were thus obtained.

As stated earlier, these estimates of uncertainties are the result of subjective judgments as well as objective estimates. For the great preponderance of data presented in this paper, these judgments have a minor effect on the relative humidity values as well as the total uncertainty, as was shown by the small difference obtained for the three different methods of weighting.
